# Effectiveness of transcatheter arterial embolization for patients with shock from abdominopelvic trauma: A retrospective cohort study

**DOI:** 10.1016/j.amsu.2020.04.029

**Published:** 2020-05-16

**Authors:** Thana Boonsinsukh, Panitpong Maroongroge

**Affiliations:** a Department of Surgery, Faculty of Medicine, Srinakharinwirot University, Ongkharak, Nakhon Nayok, 26120, Thailand; b Department of Radiology, Faculty of Medicine, Srinakharinwirot University, Ongkharak, Nakhon Nayok, 26120, Thailand

**Keywords:** Transcatheter arterial embolization, Abdominopelvic trauma, Hypovolemic shock

## Abstract

**Background:**

Transcatheter arterial embolization (TAE) is a useful endovascular technique for controlling hemorrhage in blunt abdominopelvic trauma without shock. However, several studies have reported that TAE is safe and effective for controlling hemorrhage in hypovolemic shock.

**Objective:**

To evaluate the effectiveness of TAE for patients with shock from abdominopelvic trauma.

**Method:**

The medical records of patients with abdominopelvic trauma at Her Royal Highness Princess Maha Chakri Sirindhorn Medical Center, Srinakharinwirot University from January 2014 to January 2019 were retrospectively reviewed. We enrolled patients with shock caused by injury to solid organs or pelvic fractures who underwent TAE.

**Result:**

Of the 320 patients, 14 patients with shock underwent TAE. A total of 78.6% were male. The mean age was 37.5 years. The average injury severity score was 31.3. The most common mechanism of injury was traffic accidents (85.7%). Embolization was performed for 8 liver injuries, 5 pelvic fractures and 1 splenic injury. The treatment time for TAE was approximately 47.9 ± 33.2 min. The mean length of hospital stay was 21.3 ± 15.9 days. Two patients died (14.3%). There were no embolization-related complications. A significant improvement in systolic blood pressure (p = 0.028) and a decrease in heart rate (p = 0.001), lactate concentration (p = 0.011), and crystalloid fluid (p = 0.001) and blood transfusion requirements (p = 0.002) were observed after TAE.

**Conclusions:**

TAE is a safe and effective method for treating shock patients with a rapid or transient response to resuscitation. For patients who are nonresponsive to resuscitation, TAE is an additional useful option for arterial hemorrhage control in abdominopelvic trauma.

## Highlights

• Transcatheter arterial embolization (TAE) is a safe and effective method for treating shock patients.
• TAE is addition useful option for decrease crystalloid fluid and blood transfusion requirement.
• Complication-related TAE was rare.

## 1 Introduction

Massive bleeding resulting from abdominopelvic trauma is one of the leading causes of death across all ages [1]. Stopping bleeding can save lives and reduce associated morbidity. Surgery is often considered the definitive treatment for stopping bleeding. However, it may not always be the optimal solution for controlling bleeding in some conditions, such as arterial bleeding arising from pelvic fracture and solid organ injuries [2].

Transcatheter arterial embolization (TAE) is a useful endovascular technique for the treatment of traumatic injuries. TAE can stop bleeding in a minimally invasive manner with less disruption of normal tissue than open surgery. In addition, TAE maintains the natural tamponade effect of closed space bleeding that is lost during open surgery. Therefore, TAE is the standard treatment for controlling hemorrhage in blunt abdominopelvic trauma without shock and signs of peritonitis [3,4]. In hypovolemic shock patients, open surgery is still considered the gold standard treatment for abdominopelvic trauma [5]. However, several studies have reported that TAE is safe and effective for controlling hemorrhage in these settings [2,[6], [7], [8]].

This study aimed to evaluate the effectiveness of TAE for patients with shock from abdominopelvic trauma.

## 2 Materials and methods

The medical records of patients with abdominopelvic trauma at Her Royal Highness Princess Maha Chakri Sirindhorn Medical Center, Srinakharinwirot University from January 2014 to January 2019 were retrospectively reviewed. We enrolled patients with shock caused by injury to solid organs or pelvic fractures who underwent angiography. The exclusion criteria were patients who underwent a transcatheter angiogram but did not need embolization.

**Fig. 1:**
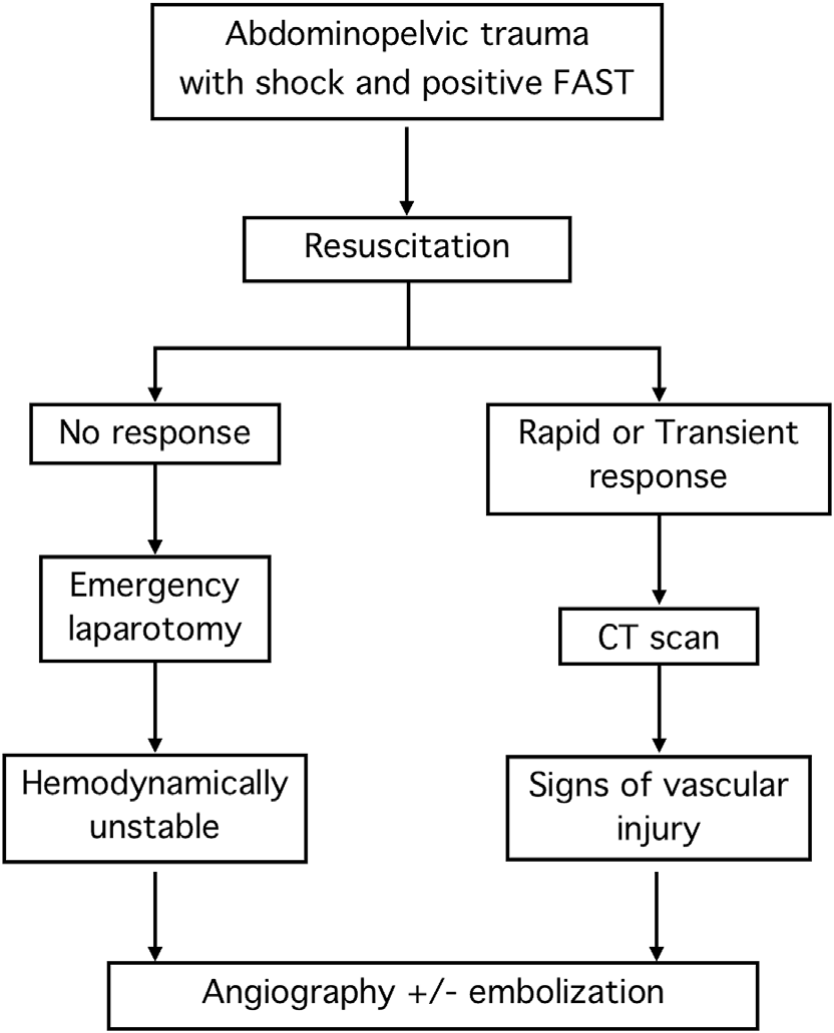
Treatment protocol for abdominopelvic trauma.

Shock was defined as a systolic blood pressure (SBP) of 90 mmHg or lower and a shock index (heart rate divided by SBP) of 1.0 or greater. The management of shock followed Advanced Trauma Life Support (ATLS) guidelines. For the patients with positive results on the Focused Assessment with Sonography for Trauma (FAST) who showed hemodynamic instability and no response to resuscitation, emergency laparotomy was performed without a computed tomography scan (CT scan). If the patients continued to be hemodynamically unstable after surgery caused by injury to solid organs or pelvic fractures, angiography and embolization were performed immediately. For hypovolemic shock patients with a rapid or transient response to resuscitation, a CT scan was performed. Angiography and embolization were performed when vascular injury caused by injury to solid organs or pelvic fractures was observed on the CT scan. The signs of vascular injury were contrast extravasation, vasospasm, pseudoaneurysm or arteriovenous fistula. The treatment protocol for abdominopelvic trauma with shock is shown in Fig. 1.

The TAE procedure was performed using the femoral puncture approach. Embolization agents included gelatin sponge pledgets (gelfoam), stainless steel coils, or both. Completion angiography was necessary to confirm the cessation of bleeding radiographically. Emergent laparotomy was considered for patients who developed shock and did not respond to fluid resuscitation during angiography.

Demographic data and outcomes were collected, including age, sex, injury characteristics, injury severity score (ISS), the time from hospital arrival to angiography, the time spent in angiography, organ of embolization, success rate of angiography, type of operation, length of hospital stay, morbidity and mortality. Pre-TAE and post-TAE data were systolic blood pressure, heart rate, base deficit, lactate concentration, and crystalloid fluid and blood transfusion requirements.

Statistical analysis was performed using SPSS (version 23) software (Statistical Procedures for Social Sciences; Chicago, Illinois, USA). Demographic data are presented as the means or median, according to the distributional characteristics of the variable. A p-value of less than 0.05 was considered statistically significant. The differences between pre- and post-TAE variables were tested using paired sample t-tests or Wilcoxon signed rank tests.

## 3 Results

During the 5-year period, 320 patients with abdominopelvic trauma were admitted. Fourteen patients with shock underwent TAE. There were 11 men and 3 women with a mean age of 37.5 ± 25.3 years. The average injury severity score (ISS) was 31.3 ± 11.3. All injuries were the result of blunt trauma. The most common mechanism of injury was traffic accidents (85.7%), and the others were falling (7.1%) and workplace accidents (7.1%). Associated injuries were as follows: 9 intrathoracic injuries, 6 intracranial injuries, 6 extremity injuries and 4 spine injuries (Table 1).

**Table 1:** Demographic and clinical characteristics of the patients (n = 14).

Characteristic	
Age (yr)	37.5 ± 25.3
Male sex (%)	78.6
ISS	31.3 ± 11.3
Mechanism (%)	
- Traffic accident - Fall - Workplace accident	85.7 7.1 7.1
Organ of embolization (%)	
- Liver injury - Pelvic injury - Splenic injury	57.1 35.8 7.1
Angiography finding (%)	
- Contrast extravasation - Abnormal contrast staining - Arteriovenous fistula	50 42.9 7.1
Associated injuries (%)	
- Intrathoracic injuries - Intracranial injuries - Extremity injuries - Spine injuries	64.3 46.1 46.1 30.8
The median time from hospital arrival to TAE (hr)	7.5 (1–65)
The average time of TAE (min)	47.9 ± 33.2
Length of ICU stay (days)	7.8 ± 7.8
Length of hospital stay (days)	21.3 ± 15.9
Survival rate (%)	85.7

ISS, injury severity score; TAE, transcatheter arterial embolization.

Embolization was performed in 8 liver injuries, 5 pelvic fractures and 1 splenic injury. Of the liver injuries, 5 received liver packing plus TAE, and 3 received only TAE. Of the pelvic fractures, 3 received pelvic external fixation with extraperitoneal pelvic packing (EPP) plus TAE, and 2 received only TAE. For the splenic injury, the patient received TAE alone. The median time from hospital arrival to TAE was 7.5 h (range, 1–65 h). The treatment time for TAE was approximately 47.9 ± 33.2 min. The most common angiographic finding was contrast extravasation (50%), and the others were abnormal contrast staining (42.9%) and arteriovenous fistula (7.1%). Embolization with gelfoam was performed in 11 patients, and embolization with gelfoam plus stainless coils was performed in 3 patients. There were no embolization-related complications, such as liver necrosis, gluteal necrosis, splenic abscess or pseudoaneurysm. None of the patients had failed angiograms.

The mean length of hospital stay was 21.3 ± 15.9 days. The mean length of ICU stay was 7.8 ± 7.8 days. Two patients died (14.3%). One 70-year-old female patient had a subdural hematoma and grade 4 liver injury. After she underwent TAE in the liver, she developed hemorrhagic shock. Unfortunately, the relatives refused surgical treatment. This patient died of uncontrollable bleeding from the liver. Another 80-year-old male patient had unstable pelvic fractures. He underwent pelvic fixation and TAE. His hemodynamics were completely stable after the operation, but he died of a heart attack 4 days later.

A significant improvement in systolic blood pressure and a decrease in heart rate, lactate concentration, and crystalloid fluid and blood transfusion requirements were observed after TAE (Table 2).

**Table 2:** Comparison between pre- and post-TAE.

Characteristic	Pre-TAE	Post-TAE	P value
Heart rate (beats/min)	119 ± 24	103 ± 14	0.001*
Systolic blood pressure (mmHg)	111 ± 24	129 ± 27	0.028*
Time adjusted PRBCs requirement (units/min)	0.022 ± 0.018	0.004 ± 0.006	0.002*
Time adjusted crystalloid fluid requirement (ml/hr)	777.6 ± 516.0	225.5 ± 162.1	0.001*
Lactate (mmol/L)	8.9 ± 6.2	5 ± 3.5	0.011*
Base-deficit (mmol/L)	−10.1 ± 7.5	−4.9 ± 4.2	0.051
Hematocrit (%)	26.9 ± 8.3	29.7 ± 5.9	0.261

TAE, transcatheter arterial embolization; PRBCs, packed red blood cells.

## 4 Discussion

The role of TAE is hemorrhagic control in hemodynamically stable patients. However, recent studies have shown successful bleeding control in hemodynamically unstable patients. Hagiwara et al. [7] reported that TAE can be performed safely for patients with blunt multiple trauma who are in hemorrhagic hypotension if their hemodynamics are improved by resuscitation with 2 L of fluid. Monnin et al. showed that TAE is very efficient and has a low complication rate in hemodynamically unstable patients with severe blunt hepatic trauma. Even in blunt splenic trauma, a study showed that TAE is a safe and effective procedure in hemodynamically unstable patients who responded to fluid resuscitation [8]. Unfortunately, Ierardi's review article reported a lack of studies to confirm that TAE is effective in hemodynamically unstable patients [2]. Our study showed that TAE can be performed in these settings. There was improvement in systolic blood pressure and a decrease in heart rate, lactate concentration, and crystalloid fluid and blood transfusion requirements. Supporting this notion, Hagiwara et al. [7] reported that fluid and blood transfusion requirements were decreased after TAE, and Cherian et al. [1] showed that TAE led to improvement in systolic blood pressure and length of ICU stay (3.5 days).

The success rate of TAE was very high. In our study, the success rate was 100%. Based on previous literature [1,2,10,11], technical success rates were 93.1% for liver injury, 94.8% for splenic injury and 98.9% for pelvic injury. The clinical success rates were 79.8% for liver injury, 84.6% for splenic injury and 91.75% for pelvic injury. Our mortality rate was 14.3%. There is no study to compare mortality rates when performing TAE and not performing TAE. However, the overall TAE study showed that the mortality rate was 8–50%.

Embolization-related complications are rare and have been reported in few studies. In hepatic embolization, gallbladder infarction following occlusion of the right hepatic artery, hepatic necrosis, liver abscess and bile leaks was reported [1,9,12]. Some studies have reported that embolization is a co-factor in liver failure [2]. Nevertheless, this was never described as a direct cause of death. In splenic embolization, there was delayed splenic rupture and recurrent bleeding. Embolization of the proximal segment of the splenic artery should be performed to reduce the pressure in the splenic parenchyma, which can help heal the laceration of the spleen [8]. Splenic infarction can occur after embolization. However, it seldom occurs because of a rich collateral network in the spleen, including the short gastric artery, gastroepiploic artery, pancreatica magna artery and dorsal pancreatic artery [13]. Other complications were splenic abscess and cyst [1,2]. In pelvic embolization, gluteal muscle necrosis, bladder or ureteral infarction and impotence were observed [14]. Nonselective embolization of the internal iliac artery, especially bilateral embolization, has higher complication rates than selective embolization. Selective embolization was recommended as a first-line treatment for pelvic trauma [15]. Our study showed no embolization-related complications.

Most studies suggested performing early TAE within 1–2 h in hemodynamically unstable patients [7,8,16]. Tanizaki et al. [17] reported that earlier embolization within 60 min had a significantly lower mortality rate than later embolization (16 vs. 64%). In our study, the median time from hospital arrival to TAE was 7.5 h because an interventional radiologist was not available all the time due to there being only one interventional radiologist in the hospital. However, our results indicated a good outcome, and Monnin et al. [9] showed that the length of hospital stay and mortality rate were not significant between early and late TAE. The hybrid trauma operating room, which is equipped with advanced medical imaging devices such as fixed C-Arms and CT scanners, is for treatments combining emergency surgery and intraoperative interventional radiologists. They can reduce the time for resuscitation and treatment to improve patient outcomes [18,19]. Kataoka et al. [20] found that hybrid treatment significantly decreased the mortality rate in severe trauma. A recent study showed a novel treatment, the Hybrid Emergency Room System (HERS) [21]. The concept of HERS is the combination of ‘‘examinations’’, “resuscitation” and ‘‘treatments’’ in the emergency room. This room can be used to perform CT scans, surgery, angiography and embolization.

This study has limitations. This was a retrospective study with a small sample size. The success rate was not dependent on TAE alone. There were other confounding factors, such as abdominal packing, pelvic external fixation and underlying disease. In our opinion, TAE alone was not the appropriate treatment in patients who were nonresponsive to resuscitation. One patient who received only TAE for a grade IV liver injury and was nonresponsive to resuscitation died. Although embolization was successful in an artery, there was still bleeding in the vein. A randomized controlled trial could confirm this result. However, this type of trial is very difficult to perform in emergency settings, and it may be unethical.

## 5 Conclusions

TAE is a safe and effective method for treating shock patients with rapid or transient responses to resuscitation. For patients who are nonresponsive to resuscitation, TAE is an additional useful option for arterial hemorrhage control in abdominopelvic trauma.

## Consent

This is a retrospective review of medical records which did not compromise patient care. Individual patient consent was not obtained. Patient's personal identification has not been disclosed anywhere in the article.

## Ethical approval

Approval was granted by Srinakharinwirot University Ethics Committee of Human Research: SWUEC/E−211/2562.

## Funding

The authors have no financial support.

## Author contribution

Thana Boonsinsukh: Data collection, data analysis, literature review, manuscript writing, patient care.

Panitpong Maroongroge: Data collection, manuscript writing, patient care.

## Research registration number

1. Name of the registry: Thai clinical trial registry
2. Unique Identifying number or registration ID: TCTR20200312005
3. Hyperlink to your specific registration (must be publicly accessible and will be checked): http://www.clinicaltrials.in.th/index.php?tp=regtrials&menu=trialsearch&smenu=fulltext&task=search&task2=view1&id=5936

## Guarantor

Thana Boonsinsukh: Corresponding author.

## Data availability

The data used to support the findings of this study are available from the corresponding author upon request.

## Disclosure

The authors had no financial support.

## Provenance and peer review

Not commissioned, externally peer reviewed.

## Declaration of competing interest

The authors declare that they have no conflicts of interest.
